# Supplementary material for: Leishmaniasis in deployed military populations: A systematic review and meta-analysis
Source: PLoS Negl Trop Dis. 2025 Mar 10;19(3):e0012680. doi: 10.1371/journal.pntd.0012680 (PMC11913291; doi:10.1371/journal.pntd.0012680)
Supplement: S1 Text — (DOCX) [file pntd.0012680.s001.docx]

**Supporting information (S1)**

**Contents:**

| 1. Search strategies | 2 |
| --- | --- |
| 1. Characteristics and quality assessment of included studies | 5 |
| - - 1. Table A: Characteristics of included studies | 5 |
| - - 1. Table B: Quality assessment using National Institutes of Health Quality Assessment Tools | 8 |
| - - 1. Table C: Quality assessment using the Newcastle Ottawa scale | 9 |
| - - 1. Table D: Adapted Newcastle-Ottawa Scale for Cohort and Case Control Studies | 10 |
| 1. Funnel plots | 11 |
| - - 1. Fig A. Funnel plot for publication bias in meta-analysis of SCL | 11 |
| - - 1. Fig B. Funnel plot for publication bias in meta-analysis of AL | 11 |
| - - 1. Fig C. Funnel plot for publication bias in meta-analysis of risk factors | 12 |
| 1. Forest plots for additional analyses | 13 |
| - - 1. Fig D. Meta-analysis of the cumulative incidence of SCL by region. | 13 |
| - - 1. Fig E. Meta-analysis of the cumulative incidence of SCL by duration of deployment | 14 |
| - - 1. Fig F. Meta-analysis of the cumulative incidence of SCL using untransformed proportions | 15 |
| - - 1. Fig G. Meta-analysis of the cumulative incidence of SCL using Logit-transformed proportions | 15 |
| - - 1. Fig H. Meta-analysis of the cumulative incidence of asymptomatic *Leishmania* infections using untransformed proportions | 16 |
| - - 1. Fig I. Meta-analysis of the cumulative incidence of asymptomatic *Leishmania* infections using Logit-transformed proportions | 16 |
| 1. Sensitivity analyses | 17 |
| - - 1. Table E: Sensitivity analyses for the meta-analysis of SCL by excluding each study | 17 |
| - - 1. Table F: Sensitivity analyses for the meta-analysis of asymptomatic leishmaniasis by excluding each study | 17 |
| - - 1. Table G: Sensitivity analyses for the meta-analysis of risk factor (Failure to regularly use insect repellent daily | 18 |
| - - 1. Table H: Sensitivity analyses for the meta-analysis of risk factor (Failure to regularly use long sleeved clothing | 18 |
| 1. PROSPERO protocol registration | 19 |
| 1. PRISMA checklist, MOOSE checklists | 23 |
| - - 1. Table I: PRISMA 2020 Checklist | 23 |
| - - 1. Table J: MOOSE Checklist for Meta-analyses of Observational Studies | 26 |
| 1. Table K: Leishmania and Sand fly species identified in the different WHO regions from the included studies | 27 |
| 1. Table L: Egger’s test of small study effect for analysis of risk factors | 28 |
| 1. References | 29 |

1. **Search strategies**

**Medline (Ovid)**

#1 ((leishmaniasis or cutaneous leishmaniasis or visceral leishmaniasis or mucocutaneous leishmaniasis) and (military personnel or soldiers or military service or armed forces)) (247)

#2 ((leishmaniasis or cutaneous leishmaniasis or visceral leishmaniasis or mucocutaneous leishmaniasis) and (military or military medicine or military health)) (304)

#3 1 or 2 (321)

#4 (("leishmaniasis" or "cutaneous leishmaniasis" or "visceral leishmaniasis" or "mucocutaneous leishmaniasis") and ("military personnel" or "military service" or "armed forces" or "soldiers") and ("incidence" or "prevalence" or "sandfly species" or "Leishmania species" or "sand fly salivary gland antigen exposure" or "risk factors")) (64)

#5 ((military or troops or army or soldiers or "armed forces") and (prevalence or exposure or causes or "risk factor" or sandfl* or vector or "causative organisms" or risks or risk or exposed or exposure* or incidence or "risk factors") and (leishmaniasis or "visceral Leishmaniasis" or leishmania or "cutaneous Leishmaniasis")) (162)

#6 3 or 4 or 5 (342)

#7 limit 6 to yr="1990 - 2022" (295)

#8 limit 7 to english language (264)

**Embase (Ovid)**

#1 ((leishmaniasis or cutaneous leishmaniasis or visceral leishmaniasis or mucocutaneous leishmaniasis) and (military personnel or soldiers or military service or armed forces)) (226)

#2 ((leishmaniasis or cutaneous leishmaniasis or visceral leishmaniasis or mucocutaneous leishmaniasis) and (military or military medicine or military health)) (314)

#3 1 or 2 (381)

#4 (("leishmaniasis" or "cutaneous leishmaniasis" or "visceral leishmaniasis" or "mucocutaneous leishmaniasis") and ("military personnel" or "military service" or "armed forces" or "soldiers") and ("incidence" or "prevalence" or "sandfly species" or "Leishmania species" or "sand fly salivary gland antigen exposure" or "risk factors")) (55)

#5 ((military or troops or army or soldiers or "armed forces") and (prevalence or exposure or causes or "risk factor" or sandfl* or vector or "causative organisms" or risks or risk or exposed or exposure* or incidence or "risk factors") and (leishmaniasis or "visceral Leishmaniasis" or leishmania or "cutaneous Leishmaniasis")) (243)

#6 3 or 4 or 5 (419)

#7 limit 6 to yr="1990 - 2022" (359)

#8 limit 7 to english language (328)

**Web of Science**

#1 leishmaniasis or cutaneous leishmaniasis or visceral leishmaniasis or mucocutaneous leishmaniasis (All Fields) and military personnel or soldiers or military service or armed forces (All Fields) (317)

#2 leishmaniasis or cutaneous leishmaniasis or visceral leishmaniasis or mucocutaneous leishmaniasis (All Fields) and military or military medicine or military health (All Fields) (394)

#3 ALL=((("leishmaniasis" or "cutaneous leishmaniasis" or "visceral leishmaniasis" or "mucocutaneous leishmaniasis") and ("military personnel" or "military service" or "armed forces" or "soldiers") and ("incidence" or "prevalence" or "sandfly species" or "Leishmania species" or "sand fly salivary gland antigen exposure" or "risk factors"))) (54)

#4 ALL=(((military or troops or army or soldiers or "armed forces") and (prevalence or exposure or causes or "risk factor" or sandfl* or vector or "causative organisms" or risks or risk or exposed or exposure* or incidence or "risk factors") and (leishmaniasis or "visceral Leishmaniasis" or leishmania or "cutaneous Leishmaniasis"))) (439)

#5 1 OR 2 OR 3 OR 4 (651)

#6 5 Timespan: 1990-01-01 to 2022-01-01 (Publication Date) (600)

#7 6 and English (Languages) (574)

**Scopus**

#1 ALL ( ( ( leishmaniasis OR cutaneous AND leishmaniasis OR visceral AND leishmaniasis OR mucocutaneous AND leishmaniasis ) AND ( military AND personnel OR soldiers OR military AND service OR armed AND forces ) ) ) (398)

#2 ALL ( ( ( leishmaniasis OR cutaneous AND leishmaniasis OR visceral AND leishmaniasis OR mucocutaneous AND leishmaniasis ) AND ( military OR military AND medicine OR military AND health ) ) ) (1907)

#3 ALL ( ( ( "leishmaniasis" OR "cutaneous leishmaniasis" OR "visceral leishmaniasis" OR "mucocutaneous leishmaniasis" ) AND ( "military personnel" OR "military service" OR "armed forces" OR "soldiers" ) AND ( "incidence" OR "prevalence" OR "sandfly species" OR "leishmania species" OR "sand fly salivary gland antigen exposure" OR "risk factors" ) ) ) (1168)

#4 ALL ( ( ( military OR troops OR army OR soldiers OR "armed forces" ) AND ( prevalence OR exposure OR causes OR "risk factor" OR sandfl* OR vector OR "causative organisms" OR risks OR risk OR exposed OR exposure* OR incidence OR "risk factors" ) AND ( leishmaniasis OR "visceral leishmaniasis" OR leishmania OR "cutaneous leishmaniasis" ) ) ) (4476)

#5 ( ALL ( ( ( leishmaniasis OR cutaneous AND leishmaniasis OR visceral AND leishmaniasis OR mucocutaneous AND leishmaniasis ) AND ( military AND personnel OR soldiers OR military AND service OR armed AND forces ) ) ) ) AND ( ALL ( ( ( leishmaniasis OR cutaneous AND leishmaniasis OR visceral AND leishmaniasis OR mucocutaneous AND leishmaniasis ) AND ( military OR military AND medicine OR military AND health ) ) ) ) AND ( ALL ( ( ( "leishmaniasis" OR "cutaneous leishmaniasis" OR "visceral leishmaniasis" OR "mucocutaneous leishmaniasis" ) AND ( "military personnel" OR "military service" OR "armed forces" OR "soldiers" ) AND ( "incidence" OR "prevalence" OR "sandfly species" OR "leishmania species" OR "sand fly salivary gland antigen exposure" OR "risk factors" ) ) ) ) AND ( ALL ( ( ( military OR troops OR army OR soldiers OR "armed forces" ) AND ( prevalence OR exposure OR causes OR "risk factor" OR sandfl* OR vector OR "causative organisms" OR risks OR risk OR exposed OR exposure* OR incidence OR "risk factors" ) AND ( leishmaniasis OR "visceral leishmaniasis" OR leishmania OR "cutaneous leishmaniasis" ) ) ) ) (194)

#6 5 AND PUBYEAR > 1989 AND PUBYEAR < 2023 (182)

#7 6 AND ( LIMIT-TO ( LANGUAGE , "english" ) ) (176

1. **Characteristics and quality assessment of included studies**
   1. **Table A. Characteristics of included studies**

| **Author and year** | **Study location** | | **Year of data collection** | **Sample size** | **Study design** | **Outcomes measured/explored** | **Diagnostic method used** | **Types of leishmaniasis** |
| --- | --- | --- | --- | --- | --- | --- | --- | --- |
|  | **Country** | **Region** |  |  |  |  |  |  |
| Andrade et al., 2005[1] | Brazil | AMR | Jul 2002 – Jun 2003 | 8,406 sandflies | Cross-Sectional Study | Characterisation of sandflies species | NOS | CL |
| Aronson et al., 2022 [2] | Iraq | EMR | 2003 – 2004 | 248 US soldiers | Case-control study | Antibody response to sand fly saliva | NOS | NOS |
| Bailey, M. S. et al., 2012 [4] | Afghanistan | EMR | Jun – Oct 2004 | 120 British soldiers | Cohort study | Cumulative incidence of leishmaniasis, characterisation of *Leishmania* species and risk factors | Histo and mole | CL |
| Bezold et al., 2001 [5] | French Guiana | AMR | Nov 1999 | 2 German soldiers (cases) | Case report | Characterisation of *Leishmania* species | Sero and mole | CL |
| Biddlestone et al., 1994 [6] | Belize | AMR | NRS | 34 British soldiers (cases) | Cohort Study | Characterisation of *Leishmania* species | Histo | CL |
| Claborn et al., 2009 [7] | Southern United States (Texas, Kentucky & North Carolina) | AMR | 2006 – 2007 | 2,042 sand flies | Cross-Sectional Study | Characterisation of sandflies species | NOS | NOS |
| Coleman et al., 2007 [8] | Iraq | EMR | Apr 2003 – Nov 2004 | 1,630 sand flies | Cross-Sectional Study | Characterisation of sandflies species | NOS | NOS |
| Correa-Cárdenas et al., 2020 [9] | Colombia | AMR | NRS | 136 Colombian patients | Cross-Sectional Study | Characterisation of *Leishmania* species. | Mole | CL |
| Dantas-Torres et al., 2017 [10] | Brazil | AMR | Jul 2012 – Jul 2014 | 24,606 sand flies  And 40 Brazilian soldiers | Cross-Sectional Study And  Retrospective cohort study | Characterisation of sand flies and *Leishmania* species. | Histo and mole | CL |
| Fryauff et al., 1993 [11] | Egypt | AFR | Jul 1989 and Jun 1991 | 8,253 sand flies  And 60 Egyptian soldiers | Cross-Sectional Study And  Retrospective cohort study | Cumulative incidence of leishmaniasis, characterisation of sand flies and *Leishmania* species | Histo | CL |
| Geraci et al., 2014 [12] | Iraq | EMR | NRS | 69 US soldiers | Case-control study | Antibody response to sand fly saliva | NOS | NOS |
| Gomes et al., 2013 [13] | Brazil | AMR | Mar 2002 – Feb 2003 | 10,762 sand flies | Cross-Sectional Study | Characterisation of sandflies species | NOS | NOS |
| González et al., 2017 [14] | Colombia | AMR | NRS | 300 Colombian soldiers | Cross-Sectional Study | Risk factors | NOS | NOS |
| Gunathilaka et al., 2020 [15] | Sri Lanka | SEAR | Sep 2018 and Aug 2019 | 5000 Sri Lankan soldiers | Cross-Sectional Study | Risk factors | NOS | NOS |
| Halsey et al., 2004 [16] | Oman | EMR | Oct 2001 – Mar 2002 | 1 US soldier | Case Report | Characterisation of *Leishmania* species | Histo, sero and mole | VL |
| Henry et al., 2021 [17] | French Guiana | AMR | Jan – Aug 2020 | 858 French soldiers  1427 French soldiers | Cohort study and a case-control study | Cumulative incidence of leishmaniasis, characterisation of *Leishmania* species and risk factors | Mole | CL |
| HEPBURN et al., 1993 [18] | Belize | AMR | 1977 – 1990 | 1800 British soldiers | Cohort study | Characterisation of *Leishmania* species | Histo | CL |
| Khan, N. H. et al., 2016 [19] | Pakistan | EMR | May 2010 – Sep 2010 | 71 Pakistani soldiers | Cross-Sectional Study | Characterisation of *Leishmania* species | Histo and mole | CL |
| Kniha et al., 2020 [20] | Kosovo | EUR | 2013 | 261 Australian soldiers | Cross-Sectional Study | Cumulative incidence of asymptomatic leishmaniasis | Sero | Asymptomatic CL and VL |
| Krüger et al., 2011 [21] | Afghanistan | EMR | 2007 – 2009 | 511 sand flies | Cross-Sectional Study | Characterisation of sandflies species | NOS | NOS |
| Lakhal-Naouar et al., 2021 [22] | Iraq | EMR | NRS | 200 US soldiers | Case-control study | Antibody response to sand fly saliva | NOS | NOS |
| Magill et al., 1993 [23] | Saudi Arabia | EMR | NRS | 8 US soldiers | Case Series | Characterisation of *Leishmania* species | Histo, sero and mole | VL |
| Matheson et al., 2012 [24] | Afghanistan | EMR | Jan 2009 | 2 British soldiers | Case series | Characterisation of *Leishmania* species | Histo and mole | CL |
| Mody et al., 2019 [26] | Iraq | EMR | 2002 – 2011 | 250 US soldiers | Case-control Study | Cumulative incidence of asymptomatic leishmaniasis, characterisation of *Leishmania* species and risk factors | Sero and mole | Asymptomatic VL |
| Myles et al., 2007 [25] | Iraq, Afghanistan | EMR | NRS | 4 US soldiers | Case series | Characterisation of *Leishmania* species | Histo, sero and mole | VL |
| Obwaller et al., 2018 [27] | Syria, Lebanon and Bosnia | EMR & EUR | Jun – Jul 2013 | 225 Australian soldiers | Cross-Sectional Study | Cumulative incidence of asymptomatic leishmaniasis, characterisation of *Leishmania* species and risk factors | Sero and mole | Asymptomatic CL and VL |
| Oré et al., 2015 [28] | Peru | AMR | May 2010 | 303 Peruvian soldiers | Cohort study | Cumulative incidence of leishmaniasis, characterisation of *Leishmania* species and risk factors | Histo and mole | CL |
| Patino et al., 2017 [29] | Colombia | AMR | 2013 | 273 Colombian soldiers | Cross-Sectional Study | characterisation of *Leishmania* species | Mole | CL |
| Royer & Crowe, 2002 [30] | Panama | AMR | NRS | 3 US soldiers (cases) | Case series | Cumulative incidence of leishmaniasis, characterisation of *Leishmania* species and risk factors | Histo | CL |
| Sanchez, J. L. et al., 1992 [31] | Panama | AMR | Jul 1984 | 540 Puerto Rican soldiers | Cross-Sectional Study | Cumulative incidence of leishmaniasis, characterisation of *Leishmania* species and risk factors | Histo | CL |
| Van Der Snoek et al., 2009 [32] | Suriname | AMR | Feb – Apr 2008 | 2 Dutch soldiers (cases) | Case series | Characterisation of *Leishmania* species and risk factors | Histo and mole | CL |
| van Thiel et al., 2010 [33] | Afghanistan | EMR | Jun –Nov 2005 | 938 Dutch soldiers | Cohort study | Cumulative incidence of leishmaniasis, characterisation of *Leishmania* species and risk factors | Histo and mole | CL |
| van Thiel et al., 2011 [34] | Belize | AMR | 28 Sep – 26 Oct 1998  29 Aug – 14 Oct 2004  31 Aug – 7 Oct 2009 | 213 Dutch soldiers | Cohort study | Cumulative incidence of leishmaniasis, characterisation of *Leishmania* species and risk factors | Histo and mole | CL |
| Vickery et al., 2008 [35] | Iraq, Kuwait and Afghanistan | EMR | Jan 2005 – May 2006 Mar 2006 – Feb 2007 | 3,446 US soldiers | Cross -sectional survey | Risk factors | NOS | NOS |
| Wijerathna et al., 2022 [36] | Sri Lanka | SEAR | Nov 2018 – Mar 2020 | 76 Sri Lankan soldiers | Cross-Sectional Study | Characterisation of *Leishmania* species | Histo and mole | CL |
| Woodrow et al., 2006 [37] | Afghanistan | EMR | NRS | 1 US soldier | Case report | Characterisation of *Leishmania* species | Sero and mole | CL and VL |

**Abbreviation:** AMR – American region; EMR – Eastern Mediterranean region; SEAR – South-East Asian region; AFR – African region; EUR – European region; NRS – Not reported in the study; NOS – Not the objective of the study; Histo – Histopathology; Sero – Serology; Mole – Molecular technic; CL – Cutaneous leishmaniasis; VL – Visceral leishmaniasis

- 1. **Table B. Quality assessment of studies included in meta-analysis using National Institutes of Health Quality Assessment Tools**

| **Study** | **Study design^δ^** | **National Institutes of Health Quality Assessment Tools question number and score allocated ^a^** | | | | | | | | | | | | | | | | |
| --- | --- | --- | --- | --- | --- | --- | --- | --- | --- | --- | --- | --- | --- | --- | --- | --- | --- | --- |
|  |  | **1** | **2** | **3** | **4** | **5** | **6** | **7** | **8** | **9** | **10** | **11** | **12** | **13** | **14** | **Rating** | **Raters initials** |  |
| Bailey et al. 2012 [4] | Cohort | Y | Y | Y | Y | N | N | Y | NA | Y | Y | Y | NA | Y | N | Good | NR & OC |  |
| Fryauff et al. 1993 [11] | Cohort | N | N | Y | Y | N | Y | Y | NA | Y | N | Y | NA | N | N | Fair | NR & OC |  |
| Henry et al. 2021 [17] | Cohort | Y | Y | Y | Y | Y | N | Y | NA | Y | Y | Y | NA | Y | Y | Good | NR & OC |  |
| Oré et al. 2015 [28] | Cohort | Y | Y | Y | Y | Y | N | Y | NA | Y | Y | Y | NA | Y | Y | Good | NR & OC |  |
| van Thiel et al. 2010 [33] | Cohort | Y | Y | Y | Y | N | N | Y | NA | Y | Y | Y | NA | Y | Y | Good | NR & OC |  |
| van Thiel et al. 2011 [34] | Cohort | Y | Y | Y | Y | Y | N | Y | NA | Y | Y | Y | NA | Y | N | Good | NR & OC |  |
| Kniha et al. 2020 [20] | Cross-Sectional | Y | Y | Y | Y | N | N | Y | Y | Y | Y | Y | NA | Y | N | Good | NR & OC |  |
| Sanchez et al. 1992 [31] | Cross-Sectional | Y | Y | Y | Y | Y | N | Y | NA | Y | N | Y | NA | NR | N | Fair | NR & MB |  |
| Obwaller et al. 2018 [27] | Cross-Sectional | Y | Y | Y | Y | N | N | Y | Y | Y | N | Y | NA | NR | N | Fair | NR & OC |  |
| Mody et al. 2019 [26] | Case-control | Y | Y | Y | N | Y | Y | N | N | Y | Y | N | NA | - | - | Fair | NR & MB |  |
| Royer & Crowe. 2002 [30] | Case series | Y | Y | Y | Y | NA | Y | Y | NA | Y | - | - | - | - | - | Good | NR & OC |  |
| **Total** | |  |  |  |  |  |  |  |  |  |  |  |  |  |  | **Fair n=4 (36.4%)**  **Good n=7 (63.6%)** |  |  |

**Abbreviations:** Y – yes, N – no, NA – not applicable, NR – not repoprted, ^a^ The National Institutes of Health (NIH) Quality Assessment Tools (<https://www.nhlbi.nih.gov/health-topics/study-quality-assessment-tools>) were utilised for evaluating the quality of the included studies [35].The rating for assessing study quality is as follows: A study was classified as "good" if the potential bias was minimal, categorised as "fair" if there was a moderate risk of bias that didn't render the results unreliable, and labelled as "poor" if there was a substantial risk of bias that could considerably impact the interpretation of the results. Reviewer’s initials relate to manuscript authors: NR – Niba Rawlings, OC – Orin Courtenay, MB – Mark Bailey.

**^δ^** All retrospective cohort studies.

- 1. **Table C. Quality assessment of cohort and case-control studies included in meta-analysis using the Newcastle Ottawa scale**

| **Study** | **Study design^δ^** | **Newcastle Ottawa Scale question number and score allocated ^a^** | | | | | | | | | | | |
| --- | --- | --- | --- | --- | --- | --- | --- | --- | --- | --- | --- | --- | --- |
|  |  | **1** | **2** | **3** | **4** | **5** | **6** | **7** | **8** | **Total stars^b^** | **Rating^c^** | **Reviewer initials^d^** |  |
| Bailey, M. S. et al., 2012 [4] | Cohort | a* | a* | a* | a* | c | b* | a* | b* | 7 | High | NR & OC |  |
| Fryauff et al., 1993 [11] | Cohort | a* | a* | a* | b | c | b* | a* | b* | 6 | Medium | NR & OC |  |
| Henry et al., 2021 [17] | Cohort | a* | a* | a* | a* | a* | b* | a* | a* | 8 | High | NR & OC |  |
| Oré et al., 2015 [28] | Cohort | a* | a* | a* | a* | a* | b* | a* | b* | 8 | High | NR & MB |  |
| van Thiel et al., 2010 [33] | Cohort | a* | a* | a* | a* | c | b* | a* | b* | 7 | High | NR & OC |  |
| van Thiel et al., 2011 [34] | Cohort | a* | a* | a* | a* | a* | b* | a* | a* | 8 | High | NR & MB |  |
| **Total** | | **n*=6**  **100%** | **n*=6**  **100%** | **n*=6**  **100%** | **n*=6**  **100%** | **n*=3**  **50%** | **n*=6**  **100%** | **n*=6**  **100%** | **n*=6**  **100%** | **Range 7-8** | **Medium n=1 (16.7%)**  **High n-5 (83.3%)** |  |  |
| Mody et al., 2019 [26] | Case-control | a* | a* | b | a* | a* | a* | a* | b | 6 | Medium | NR & OC |  |

^a^ Newcastle-Ottawa question numbers 1-8, answers and associated number of stars (*). ^b^ Minimum number of possible stars to be awarded = 0, maximum number of possible stars to be awarded = 8. ^c^ Categories were allocated as: Low = 0-3 stars, Medium = 4-6 stars, High = 7-8 stars. ^d^ Reviewers initials relate to manuscript authors: NR: Niba Rawlings, OC: Orin Courtenay, MB: Mark Bailey

**^δ^** All retrospective cohort studies.

- 1. **Table D. Adapted Newcastle-Ottawa Scale for Cohort and Case Control Studies**

| **COHORT STUDIES**  Note: A study can be awarded a maximum of one star for each numbered item within the Selection and Outcome categories. A maximum of two stars can be given for Comparability.  **Selection (select one only)**  1) Representativeness of the exposed cohort  a) truly representative of the average deployed troop population *  b) somewhat representative of the average deployed troop population *  c) selected group of users eg deployed troop population  d) no description of the derivation of the cohort  2) Selection of the non-exposed cohort  a) drawn from the same population of deployed troops as the exposed cohort *  b) drawn from a different source  c) no description of the derivation of the non-exposed cohort  3) Ascertainment of exposure  a) secure record (eg deployment records) *  b) structured interview *  c) written self-report  d) no description  4) Demonstration that outcome of interest was not present at start of study  a) yes *  b) no  **Comparability**  5) Comparability of cohorts on the basis of the design or analysis  a) study controls for multiple deployments to leishmaniasis endemic areas *  b) study controls for any additional factor*  c) no factors controlled for  **Outcome**  6) Assessment of outcome  a) independent blind assessment *  b) record linkage *  c) self-report  d) no description  7) Was follow-up long enough for outcomes to occur  a) yes (select an adequate follow up period for outcome of interest) *  b) no  8) Adequacy of follow up of cohorts  a) complete follow up - all subjects accounted for *  b) subjects lost to follow up unlikely to introduce bias - small number lost (> 80 %  follow up, or description provided of those lost) *  c) follow up rate < 80% 0r >20% and no description of those lost  d) no statement | **CASE CONTROL STUDIES**  Note: A study can be awarded a maximum of one star for each numbered item within the Selection and Exposure categories. A maximum of two stars can be given for Comparability.  **Selection**  1) Is the case definition adequate?  a) yes, with independent validation *  b) yes, eg record linkage or based on self-reports c) no description  2) Representativeness of the cases  a) consecutive or obviously representative series of cases *  b) potential for selection biases or not stated  3) Selection of Controls  a) community controls * (Same unit or regiment)  b) hospital controls (different unit or regiment within same service)  c) no description  4) Definition of Controls  a) no history of disease (endpoint) *  b) no description of source  **Comparability**  5) Comparability of cases and controls on the basis of the design or analysis  a) study controls for multiple deployments to leishmaniasis endemic areas *  b) study controls for any additional factor *  **Exposure**  6) Ascertainment of exposure  a) secure record (eg deployment records) *  b) structured interview where blind to case/control status *  c) interview not blinded to case/control status  d) written self-report or medical record only  e) no description  7) Same method of ascertainment for cases and controls  a) yes *  b) no  8) Non-Response rate  a) same rate for both groups *  b) non respondents described  c) rate different and no designation |
| --- | --- |

a- The primary exposure is leishmaniasis (cutaneous of visceral)

1. **Funnel plots**
   1. **Fig A. Funnel plot for publication bias in meta-analysis of symptomatic CL**

- 1. **Fig B. Funnel plot for publication bias in meta-analysis of asymptomatic *Leishmania* infections**

- 1. **Fig C. Funnel plot for publication bias in meta-analysis of risk factors**

1. **Forest plots for additional analyses**
   1. **Fig D. Meta-analysis of the cumulative incidence of symptomatic CL by region.**

Note: *Different data presented in the same study

References: Bailey et al. 2012 [4], Fryauff et al. 1993 [11], Henry et al. 2021 [17], Oré et al. 2015 [28], Royer & Crowe. 2002 [30], van Thiel et al. 2010 [33], van Thiel et al. 2011 [34] & Sanchez et al. 1992 [31]

- 1. **Fig E. Meta-analysis of the cumulative incidence of symptomatic CL by duration of deployment**

References: Bailey et al. 2012 [4], Fryauff et al. 1993 [11], Henry et al. 2021 [17], Oré et al. 2015 [28], Royer & Crowe. 2002 [30], van Thiel et al. 2010 [33], van Thiel et al. 2011 [34] & Sanchez et al. 1992 [31]

- 1. **Fig F. Meta-analysis of the cumulative incidence of symptomatic CL using untransformed proportions**

References: Bailey et al. 2012 [4], Fryauff et al. 1993 [11], Henry et al. 2021 [17], Oré et al. 2015 [28], Royer & Crowe. 2002 [30], Sanchez et al. 1992 [31], van Thiel et al. 2010 [33] & van Thiel et al. 2011 [34].

- 1. **Fig G. Meta-analysis of the cumulative incidence of symptomatic CL using Logit-transformed proportions**

References: Bailey et al. 2012 [4], Fryauff et al. 1993 [11], Henry et al. 2021 [17], Oré et al. 2015 [28], Royer & Crowe. 2002 [30], Sanchez et al. 1992 [31], van Thiel et al. 2010 [33] & van Thiel et al. 2011 [34].

- 1. **Fig H. Meta-analysis of the cumulative incidence of asymptomatic *Leishmania* infections using untransformed proportions**

References: Kniha et al. 2020 [20], Mody et al. 2019 [26] & Obwaller et al. 2018 [27].

- 1. **Fig I. Meta-analysis of the cumulative incidence of asymptomatic *Leishmania* infections using Logit-transformed proportions**

References: Kniha et al. 2020 [20], Mody et al. 2019 [26] & Obwaller et al. 2018 [27].

1. **Sensitivity analyses**
   1. **Table E. Sensitivity analyses for the meta-analysis of SCL by excluding each study**

| **Omitted study** | **Result of sensitivity analysis** | | **p-value** |
| --- | --- | --- | --- |
|  | **Overall pooled estimate (%)** | **95% CI (%)** |  |
| Bailey et al. (2012) | 09 | 5 – 16 | <0.001 |
| Fryauff et al. (1993) | 10 | 5 – 16 | <0.001 |
| Fryauff et al. (1993)* | 11 | 6 – 17 | <0.001 |
| Henry et al. (2021) | 11 | 6 – 17 | <0.001 |
| Henry et al. (2021)* | 11 | 6 – 17 | <0.001 |
| Ore et al. (2015) | 09 | 4 – 14 | <0.001 |
| Royer & Crowe (2002) | 11 | 5 – 17 | <0.001 |
| Sanchez et al. (1992) | 11 | 6 – 17 | <0.001 |
| van Thiel et al. (2010) | 10 | 5 – 16 | <0.001 |
| van Thiel et al. (2011) | 09 | 4 – 15 | <0.001 |
| van Thiel et al. (2011)* | 09 | 5 – 16 | <0.001 |
| Main analysis | 10 | 5 – 16 | <0.001 |

References: Bailey et al. 2012 [4], Fryauff et al. 1993 [11], Henry et al. 2021 [17], Oré et al. 2015 [28], Royer & Crowe. 2002 [30], Sanchez et al. 1992 [31], van Thiel et al. 2010 [33] & van Thiel et al. 2011 [34].

- 1. **Table F. Sensitivity analyses for the meta-analysis of asymptomatic leishmaniasis by excluding each study**

| **Omitted study** | **Result of sensitivity analysis** | | **p-value** |
| --- | --- | --- | --- |
|  | **Overall pooled estimate (%)** | **95% CI (%)** |  |
| Kniha et al. (2020) | 10 | 4 – 18 | 0.005 |
| Mody et al, (2019) | 9 | 5 – 14 | 0.001 |
| Obwaller et al. (2018) | 13 | 9 – 19 | <0.001 |
| Obwaller et al. (2018)* | 12 | 6 – 19 | <0.001 |
| Obwaller et al. (2018)** | 11 | 5 – 19 | 0.003 |
| Main analysis | 11 | 6 – 17 | <0.001 |

References: Kniha et al. 2020 [20], Mody et al. 2019 [26] & Obwaller et al. 2018 [27].

- 1. **Table G. Sensitivity analyses for the meta-analysis of risk factor (Failure to regularly use insect repellent daily)**

| **Omitted study** | **Result of sensitivity analysis** | | **p-value** |
| --- | --- | --- | --- |
|  | **Overall pooled estimate (%)** | **95% CI (%)** |  |
| Gunathilaka et al. (2020) | 0.14 | -0.20 – 0.47 | 0.422 |
| Henry et al, (2021) | 0.43 | -0.36 – 1.23 | 0.287 |
| Mody et al. (2019) | 0.36 | -0.43 – 1.15 | 0.371 |
| Van Thiel et al. (2010)** | 0.46 | -0.35 – 1.26 | 0.264 |
| Vickery et al. (2008)* | 0.56 | -0.06 – 1.19 | 0.075 |
| Main analysis | 0.40 | -0.23 – 1.03 | 0.210 |

References: Gunathilaka et al., 2020 [15], Henry et al. 2021 [17], Mody et al. 2019 [26], van Thiel et al. 2010 [33] & Vickery et al., 2008 [35].

- 1. **Table H. Sensitivity analyses for the meta-analysis of risk factor (Failure to regularly use long sleeved clothing)**

| **Omitted study** | **Result of sensitivity analysis** | | **p-value** |
| --- | --- | --- | --- |
|  | **Overall pooled estimate (%)** | **95% CI (%)** |  |
| Gunathilaka et al. (2020)** | 0.50 | 0.22 – 0.79 | 0.001 |
| Henry et al. (2021)** | 0.81 | 0.36 – 1.26 | <0.001 |
| Mody et al. (2019)* | 0.71 | 0.14 – 1.28 | 0.014 |
| Ore et al. (2015)* | 0.66 | 0.06 – 1.26 | 0.032 |
| van Thiel et al. (2010) | 0.85 | 0.34 – 1.36 | 0.001 |
| Main analysis | 0.72 | 0.26 – 1.17 | <0.001 |

References: Gunathilaka et al., 2020 [15], Henry et al. 2021 [17], Mody et al. 2019 [26], Oré et al. 2015 [28] & van Thiel et al. 2010 [33].

1. **PROSPERO protocol registration**


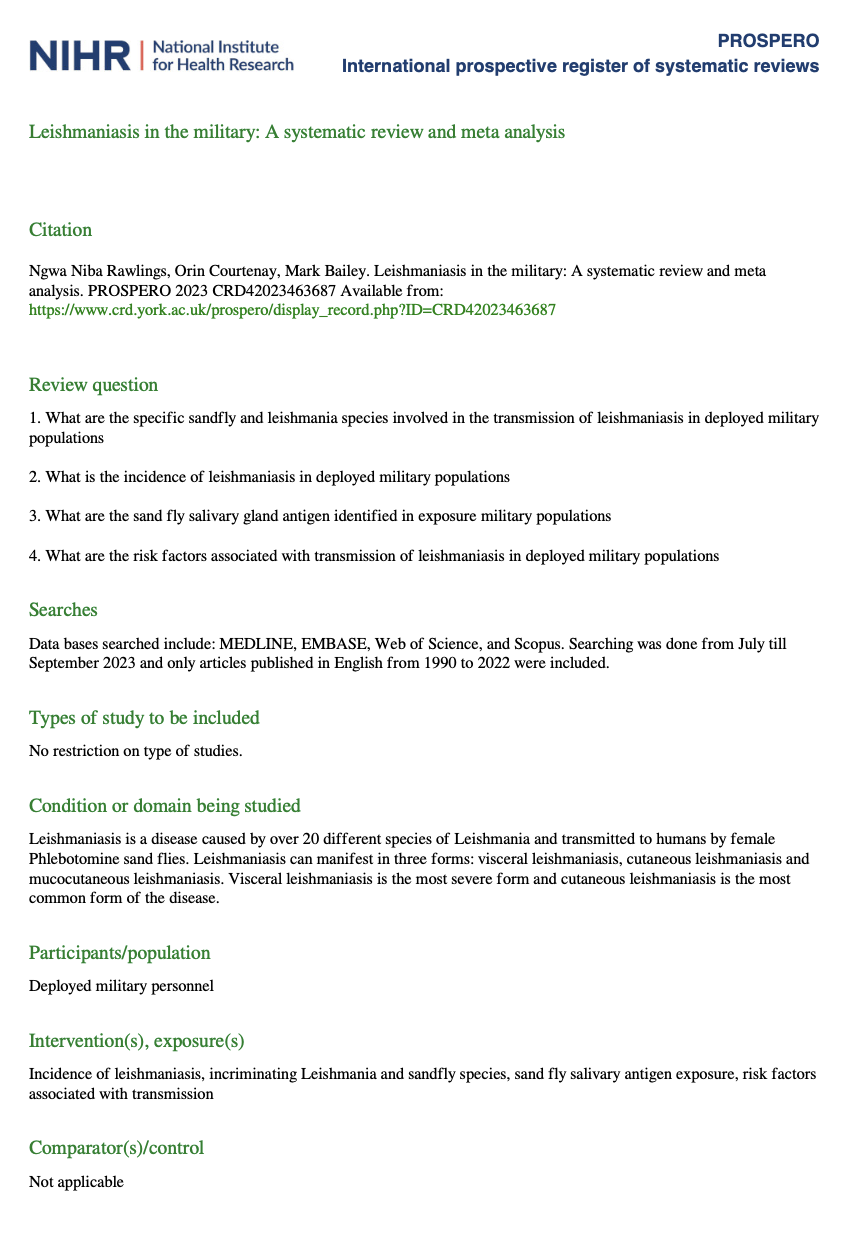


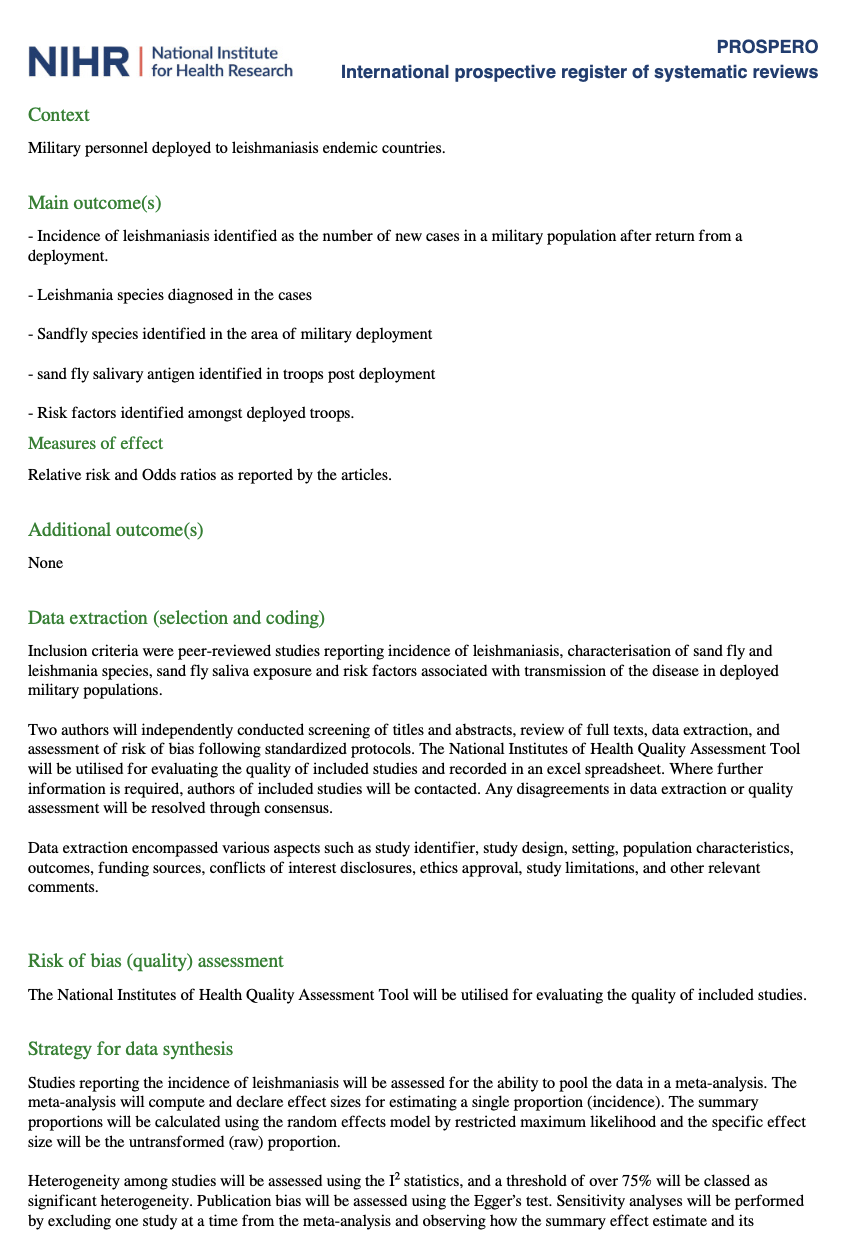


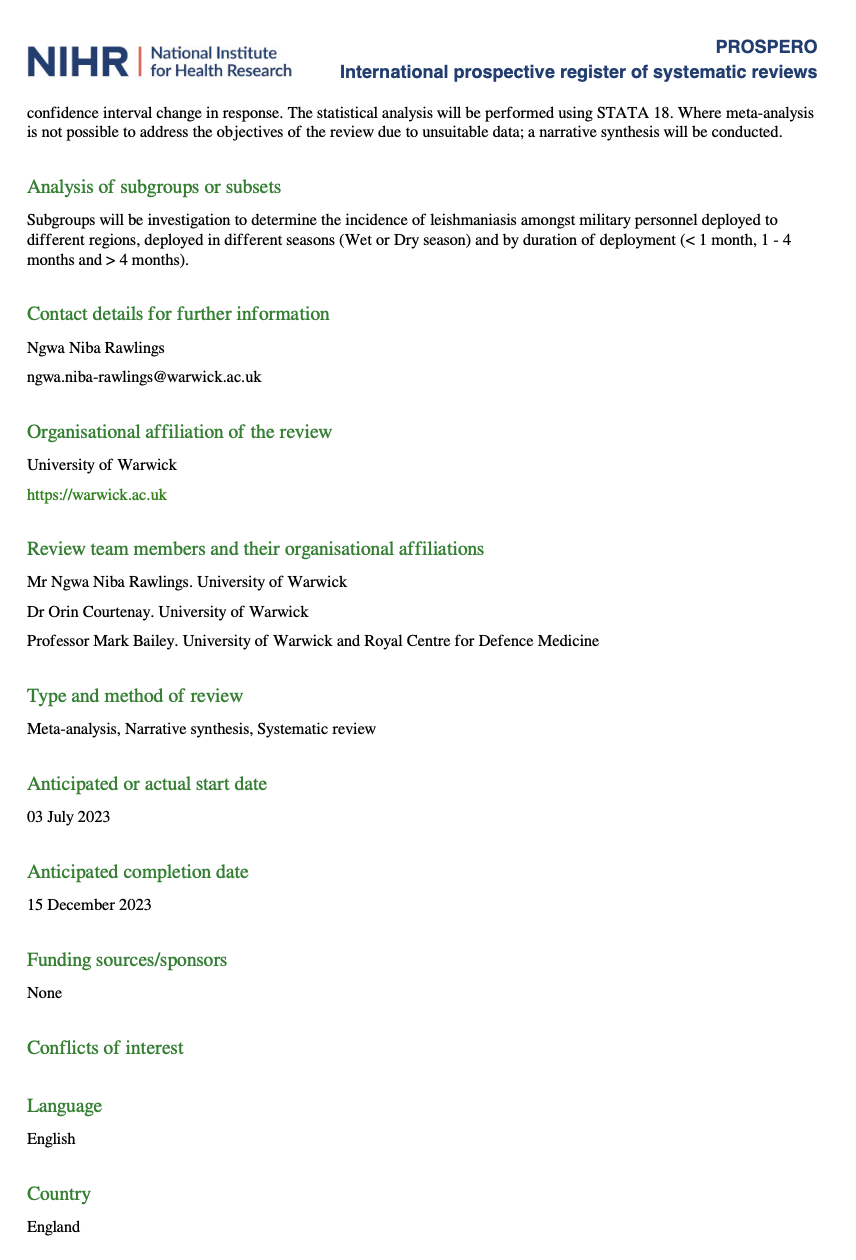


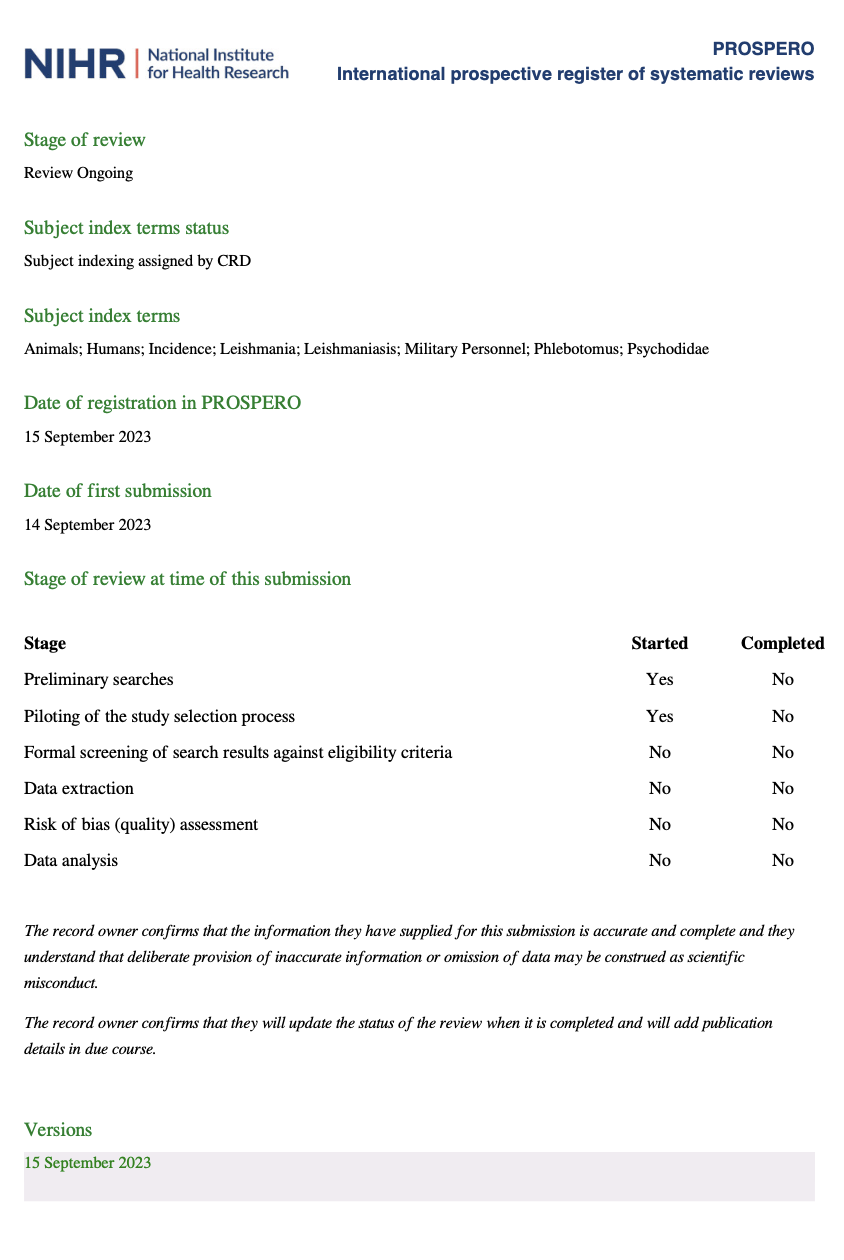


1. **PRISMA checklist, MOOSE checklists**
   1. **Table I. PRISMA 2020 Checklist** [39,40]

| **Section and Topic** | **Item #** | **Checklist item** | **Location where item is reported** |
| --- | --- | --- | --- |
| **TITLE** | | |  |
| Title | 1 | Identify the report as a systematic review. | 1 |
| **ABSTRACT** | | |  |
| Abstract | 2 | See the PRISMA 2020 for Abstracts checklist. | 1 |
| **INTRODUCTION** | | |  |
| Rationale | 3 | Describe the rationale for the review in the context of existing knowledge. | 2-4 |
| Objectives | 4 | Provide an explicit statement of the objective(s) or question(s) the review addresses. | 4 |
| **METHODS** | | |  |
| Eligibility criteria | 5 | Specify the inclusion and exclusion criteria for the review and how studies were grouped for the syntheses. | 5 |
| Information sources | 6 | Specify all databases, registers, websites, organisations, reference lists and other sources searched or consulted to identify studies. Specify the date when each source was last searched or consulted. | 5 |
| Search strategy | 7 | Present the full search strategies for all databases, registers and websites, including any filters and limits used. | 5 |
| Selection process | 8 | Specify the methods used to decide whether a study met the inclusion criteria of the review, including how many reviewers screened each record and each report retrieved, whether they worked independently, and if applicable, details of automation tools used in the process. | 5 |
| Data collection process | 9 | Specify the methods used to collect data from reports, including how many reviewers collected data from each report, whether they worked independently, any processes for obtaining or confirming data from study investigators, and if applicable, details of automation tools used in the process. | 5 |
| Data items | 10a | List and define all outcomes for which data were sought. Specify whether all results that were compatible with each outcome domain in each study were sought (e.g. for all measures, time points, analyses), and if not, the methods used to decide which results to collect. | 5 |
|  | 10b | List and define all other variables for which data were sought (e.g. participant and intervention characteristics, funding sources). Describe any assumptions made about any missing or unclear information. | 5 |
| Study risk of bias assessment | 11 | Specify the methods used to assess risk of bias in the included studies, including details of the tool(s) used, how many reviewers assessed each study and whether they worked independently, and if applicable, details of automation tools used in the process. | 5 |
| Effect measures | 12 | Specify for each outcome the effect measure(s) (e.g. risk ratio, mean difference) used in the synthesis or presentation of results. | 5-6 |
| Synthesis methods | 13a | Describe the processes used to decide which studies were eligible for each synthesis (e.g. tabulating the study intervention characteristics and comparing against the planned groups for each synthesis (item #5)). | Table A |
|  | 13b | Describe any methods required to prepare the data for presentation or synthesis, such as handling of missing summary statistics, or data conversions. | 5-6 |
|  | 13c | Describe any methods used to tabulate or visually display results of individual studies and syntheses. | 6 |
|  | 13d | Describe any methods used to synthesize results and provide a rationale for the choice(s). If meta-analysis was performed, describe the model(s), method(s) to identify the presence and extent of statistical heterogeneity, and software package(s) used. | 6 |
|  | 13e | Describe any methods used to explore possible causes of heterogeneity among study results (e.g. subgroup analysis, meta-regression). | 6 |
|  | 13f | Describe any sensitivity analyses conducted to assess robustness of the synthesized results. | 6 |
| Reporting bias assessment | 14 | Describe any methods used to assess risk of bias due to missing results in a synthesis (arising from reporting biases). | 5-6 |
| Certainty assessment | 15 | Describe any methods used to assess certainty (or confidence) in the body of evidence for an outcome. | 6 |
| **RESULTS** | | |  |
| Study selection | 16a | Describe the results of the search and selection process, from the number of records identified in the search to the number of studies included in the review, ideally using a flow diagram. | 6-7, Fig 1 |
|  | 16b | Cite studies that might appear to meet the inclusion criteria, but which were excluded, and explain why they were excluded. | 7, Fig 1 |
| Study characteristics | 17 | Cite each included study and present its characteristics. | 6-8, Fig1, Table A in S1 Text |
| Risk of bias in studies | 18 | Present assessments of risk of bias for each included study. | Table A in S1 Text |
| Results of individual studies | 19 | For all outcomes, present, for each study: (a) summary statistics for each group (where appropriate) and (b) an effect estimate and its precision (e.g. confidence/credible interval), ideally using structured tables or plots. | 8-15 |
| Results of syntheses | 20a | For each synthesis, briefly summarise the characteristics and risk of bias among contributing studies. | Table A in S1 Text |
|  | 20b | Present results of all statistical syntheses conducted. If meta-analysis was done, present for each the summary estimate and its precision (e.g. confidence/credible interval) and measures of statistical heterogeneity. If comparing groups, describe the direction of the effect. | 8-11, Fig 2-4, Fig A-E in S1 Text |
|  | 20c | Present results of all investigations of possible causes of heterogeneity among study results. | 11 |
|  | 20d | Present results of all sensitivity analyses conducted to assess the robustness of the synthesized results. | 11, Table B in S1 Text |
| Reporting biases | 21 | Present assessments of risk of bias due to missing results (arising from reporting biases) for each synthesis assessed. | - |
| Certainty of evidence | 22 | Present assessments of certainty (or confidence) in the body of evidence for each outcome assessed. | 8-11, Fig 2-4, Fig A,B & Table B in S1 Text |
| **DISCUSSION** | | |  |
| Discussion | 23a | Provide a general interpretation of the results in the context of other evidence. | 16-19 |
|  | 23b | Discuss any limitations of the evidence included in the review. | 18-19 |
|  | 23c | Discuss any limitations of the review processes used. | 18-19 |
|  | 23d | Discuss implications of the results for practice, policy, and future research. | 19 |
| **OTHER INFORMATION** | | |  |
| Registration and protocol | 24a | Provide registration information for the review, including register name and registration number, or state that the review was not registered. | 5 & 6 in S1 Text |
|  | 24b | Indicate where the review protocol can be accessed, or state that a protocol was not prepared. | 6 in S1 Text |
|  | 24c | Describe and explain any amendments to information provided at registration or in the protocol. | - |
| Support | 25 | Describe sources of financial or non-financial support for the review, and the role of the funders or sponsors in the review. | 19 |
| Competing interests | 26 | Declare any competing interests of review authors. | 19 |
| Availability of data, code and other materials | 27 | Report which of the following are publicly available and where they can be found: template data collection forms; data extracted from included studies; data used for all analyses; analytic code; any other materials used in the review. | - |

- 1. **Table J. MOOSE Checklist for Meta-analyses of Observational Studies** [41]

| **Item No** | **Recommendation** | **Reported on Page No** |
| --- | --- | --- |
| Reporting of background should include | | |
| 1 | Problem definition | 2-4 |
| 2 | Hypothesis statement | 4 |
| 3 | Description of study outcome(s) | 5 |
| 4 | Type of exposure or intervention used | 4-5 |
| 5 | Type of study designs used | 5 |
| 6 | Study population | 4-5 |
| Reporting of search strategy should include | | |
| 7 | Qualifications of searchers (eg, librarians and investigators) | 5 |
| 8 | Search strategy, including time period included in the synthesis and key words | 5 |
| 9 | Effort to include all available studies, including contact with authors | 5 |
| 10 | Databases and registries searched | 6, S |
| 11 | Search software used, name and version, including special features used (eg, explosion) | 5 |
| 12 | Use of hand searching (eg, reference lists of obtained articles) | 5 |
| 13 | List of citations located and those excluded, including justification | 6, Fig 1 |
| 14 | Method of addressing articles published in languages other than English | N/A |
| 15 | Method of handling abstracts and unpublished studies | 5 |
| 16 | Description of any contact with authors | N/A |
| Reporting of methods should include | | |
| 17 | Description of relevance or appropriateness of studies assembled for assessing the hypothesis to be tested | 5-6 |
| 18 | Rationale for the selection and coding of data (eg, sound clinical principles or convenience) | 5-6 |
| 19 | Documentation of how data were classified and coded (eg, multiple raters, blinding and interrater reliability) | 5-6 |
| 20 | Assessment of confounding (eg, comparability of cases and controls in studies where appropriate) | 6 |
| 21 | Assessment of study quality, including blinding of quality assessors, stratification or regression on possible predictors of study results | 5-6 |
| 22 | Assessment of heterogeneity | 6 |
| 23 | Description of statistical methods (eg, complete description of fixed or random effects models, justification of whether the chosen models account for predictors of study results, dose-response models, or cumulative meta-analysis) in sufficient detail to be replicated | 6 |
| 24 | Provision of appropriate tables and graphics | Figs 1, Fig 2-4, Fig A-E & Table A-B in S1 Text |
| Reporting of results should include | | |
| 25 | Graphic summarizing individual study estimates and overall estimate | Figs 2-4 |
| 26 | Table giving descriptive information for each study included | Table A in S1 Text |
| 27 | Results of sensitivity testing (eg, subgroup analysis) | Fig 3, Table B in S1 Text |
| 28 | Indication of statistical uncertainty of findings | 11 |

1. **Table K.** *Leishmania* and Sand fly species identified in the different WHO regions from the included studies

| **WHO region** | ***Leishmania* species** | **Sand fly species** |
| --- | --- | --- |
| American region (AMR) | *L. braziliensis** , *L. Mexicana,* *L. (V.) guyanensis*, *L. panamensis, L. naiffi, L. lindenbergi, L. infantum* and *L. lainsoni* | *Brumptomyia pintoi, Lutzomyia (Lu) abonnenci, Lu. abunaensis, Lu. amazonensis*, Lu. anduzei*, Lu. anthophora*, Lu. antunesi, Lu. aquilonia, Lu. aragaoi, Lu. ayrozai, Lu. barrettoi, Lu. bispinosa, Lu. bourrouli, Lu. braziliensis, Lu. bursiformis, Lu. capixaba, Lu. choti*, Lu. claustrei, Lu. complexa*, Lu. corossoniensis, Lu. cultellata, Lu. cuzquena, Lu. damascenoi, Lu. davisi, Lu. dendrophyla, Lu. diabolica*, Lu. dreisbachi, Lu. eurypyga, Lu. evandroi, Lu. flaviscutellata*, Lu. furcate, Lu. geniculate, Lu. georgii, Lu. hirsute, Lu. inflata, Lu. inpai, Lu. longispina*, Lu. lutziana, Lu. migonei, Lu. monstruosa, Lu. naftalekatzi, Lu. nematoducta, Lu. olmeca nociva*, Lu. oswaldoi, Lu. pacae, Lu. paraensis, Lu. pennyi, Lu. Pilosa, Lu. quinquerfer, Lu. ratcliffei, Lu. rorotaensis, Lu. ruii*, Lu. saulensis, Lu. scaffi, Lu. schreiberi, Lu. sericea, Lu. servulolimai, Lu. shannoni*, Lu. sordelli*, Lu. spathotrichia, Lu. squamiventris, Lu. tarapacaensis, Lu. triacantha, Lu. trichopyga, Lu. trispinosa, Lu. tuberculate, Lu. tupynambaí, Lu. ubiquitalis, Lu. umbratilis*, Lu. vexator*, Lu. viannamartinsi, Lu. walker*, Lu. wellcomei*, Lu. whitmani, Lu. williamsi and Lu. yuilli pajoti* |
| Eastern Mediterranean region (EMR) | *L. major**, *L. infantum*, *L. tropica*, and *L. donovani* | *Phlebotomus papatasi*, Ph. Sergenti, Ph. Alexandri*, Ph. Caucasicus, Ph.turanicus, Ph. Keshishiani, Se. murgabiensis, Se. dreyfussi turkestanica, Se. clydei and Se. grekovi* |
| European region | *L. donovani/infantum complex* |  |
| South-East Asian region SEAR | *L donovani* | No studies found |
| African region (AFR) | *L. major* | *Ph. Papatasi* and Se. antennata* |

**Note:** * represents the most reported species in the respective regions according to the included studies.

1. **Table L. Egger’s test of small study effect for analysis of risk factors**

| **Risk factor** | **z-value** | **p-value** |
| --- | --- | --- |
| Failure to regularly use insect repellent daily | -1.77 | 0.077 |
| Failure to regularly use insecticide-treated bed net | 0.20 | 0.838 |
| Failure to regularly use long sleeved clothing | -1.38 | 0.169 |
| Failure to use permethrin-treated uniform | 1.14 | 0.254 |
| Lack of knowledge about leishmaniasis | Not applicable due to limited number of studies | |
| Sleeping accommodation with easy access to vectors | Not applicable due to limited number of studies | |

1. **References**

1. Andrade MS, Valenca HF, da Silva AL, Almeida Fde A, Almeida EL, de Brito ME, et al. Sandfly fauna in a military training area endemic for American tegumentary leishmaniasis in the Atlantic Rain Forest region of Pernambuco, Brazil. Cadernos de Saude Publica. 2005;21(6):1761–7.

2. Aronson NE, Oliveira F, Gomes R, Porter WD, Howard RS, Kamhawi S, et al. Antibody Responses to Phlebotomus papatasi Saliva in American Soldiers With Cutaneous Leishmaniasis Versus Controls. Frontiers in Tropical Diseases [Internet]. 2022 [cited 2023 Aug 16];2. Available from: https://www.frontiersin.org/articles/10.3389/fitd.2021.766273

3. Aronson NE. Leishmaniasis in American Soldiers: Parasites from the Front. In: Emerging Infections 7 [Internet]. John Wiley & Sons, Ltd; 2006 [cited 2024 Apr 8]. p. 325–42. Available from: https://onlinelibrary.wiley.com/doi/abs/10.1128/9781555815585.ch17

4. Bailey MS, Caddy AJ, McKinnon KA, Fogg LF, Roscoe M, Bailey JW, et al. Outbreak of Zoonotic Cutaneous Leishmaniasis with Local Dissemination in Balkh, Afghanistan. BMJ Military Health. 2012 Sep 1;158(3):225–8.

5. Bezold G, Lange M, Gethöffer K, Pillekamp H, Reindl H, Richter C, et al. Competitive Polymerase Chain Reaction Used to Diagnose Cutaneous Leishmaniasis in German Soldiers Infected During Military Exercises in French Guiana. EJCMID. 2001 Jun 1;20(6):421–4.

6. Biddlestone LR, Hepburn NC, McLaren KM. A clinico-pathological study of cutaneous leishmaniasis in British troops from Belize. Transactions of the Royal Society of Tropical Medicine and Hygiene. 1994 Nov 1;88(6):672–6.

7. Claborn DM, Rowton ED, Lawyer PG, Brown GC, Keep LW. Species Diversity and Relative Abundance of Phlebotomine Sand Flies (Diptera: Psychodidae) on Three Army Installations in the Southern United States and Susceptibility of a Domestic Sand Fly to Infection With Old World *Leishmania major*. Military Medicine. 2009 Nov;174(11):1203–8.

8. Coleman RE, Burkett DA, Sherwood V, Caci J, Spradling S, Jennings BT, et al. Impact of Phlebotomine Sand Flies on U.S. Military Operations at Tallil Air Base, Iraq: 2. Temporal and Geographic Distribution of Sand Flies. Journal of Medical Entomology. 2007 Jan 1;44(1):29–41.

9. Correa-Cárdenas CA, Pérez J, Patino LH, Ramírez JD, Duque MC, Romero Y, et al. Distribution, treatment outcome and genetic diversity of Leishmania species in military personnel from Colombia with cutaneous leishmaniasis. BMC Infectious Diseases. 2020 Dec 9;20(1):938.

10. Dantas-Torres F, Sales KG da S, Miranda DE de O, Silva FJ da, Figueredo LA, Melo FL de, et al. Sand fly population dynamics and cutaneous leishmaniasis among soldiers in an Atlantic forest remnant in northeastern Brazil. PLOS Neglected Tropical Diseases. 2017 Feb 27;11(2):e0005406.

11. FRYAUFF DJ, MODI GB, MANSOUR. NS, KREUTZER RD, SOLIMAN S, YOUSSEF FG. Epidemiology of Cutaneous Leishmaniasis at a Focus Monitored by the Multinational Force and Observers in the Northeastern Sinai Desert of Egypt [Internet]. 1993 [cited 2023 Aug 14]. Available from: https://apps.dtic.mil/sti/citations/ADA282056

12. Geraci NS, Mukbel RM, Kemp MT, Wadsworth MN, Lesho E, Stayback GM, et al. Profiling of Human Acquired Immunity Against the Salivary Proteins of Phlebotomus papatasi Reveals Clusters of Differential Immunoreactivity. American Journal of Tropical Medicine and Hygiene. 2014 May;90(5):923–38.

13. Gomes LHM, Albuquerque MIC, Rocha LC da, Pinheiro FG, Franco AMR. Diversity and distribution of sandflies (Diptera: Psychodidae: Phlebotominae) in a military area in the state of Amazonas, Brazil. Mem Inst Oswaldo Cruz. 2013 Aug;108:651–6.

14. González AM, Solís-Soto MT, Radon K. Leishmaniasis: Who Uses Personal Protection among Military Personnel in Colombia? Annals of Global Health. 2017 May 1;83(3):519–23.

15. Gunathilaka N, Semege S, Pathirana N, Manamperi N, Udayanga L, Wijesinghe H, et al. Prevalence of cutaneous leishmaniasis infection and clinico-epidemiological patterns among military personnel in Mullaitivu and Kilinochchi districts of the Northern Province, early war-torn areas in Sri Lanka. Parasites Vectors. 2020 May 19;13(1):263.

16. Halsey ES, Bryce LM, Wortmann GW, Weina PJ, Ryan JR, DeWitt CC. Visceral leishmaniasis in a soldier returning from Operation Enduring Freedom. Military Medicine. 2004;169(9):699–701.

17. Henry K, Mayet A, Hernandez M, Frechard G, Blanc PA, Schmitt M, et al. Outbreak of Cutaneous Leishmaniasis among military personnel in French Guiana, 2020: Clinical, phylogenetic, individual and environmental aspects. PLOS Neglected Tropical Diseases. 2021 Nov 19;15(11):e0009938.

18. Hepburn NC, Tidman MJ, Hunter JAA. Cutaneous leishmaniasis in British troops from Belize. Br J Dermatol. 1993 Jan;128(1):63–8.

19. Khan NH, Bari AU, Hashim R, Khan I, Muneer A, Shah A, et al. Cutaneous Leishmaniasis in Khyber Pakhtunkhwa Province of Pakistan: Clinical Diversity and Species-Level Diagnosis. American Journal of Tropical Medicine and Hygiene. 2016 Nov;95(5):1106–14.

20. Kniha E, Walochnik J, Poeppl W, Mooseder G, Obwaller AG. Leishmania spp. seropositivity in Austrian soldiers returning from the Kosovo. Wien Klin Wochenschr. 2020 Jan 1;132(1):47–9.

21. Kruger A, Struven L, Post RJ, Faulde M. The sandflies (Diptera: Psychodidae, Phlebotominae) in military camps in northern Afghanistan (2007-2009), as identified by morphology and DNA ‘barcoding’. Annals of Tropical Medicine and Parasitology. 2011 Mar;105(2):163–76.

22. Lakhal-Naouar I, Mukbel R, DeFraites RF, Mody RM, Massoud LN, Shaw D, et al. The human immune response to saliva of Phlebotomus alexandri, the vector of visceral leishmaniasis in Iraq, and its relationship to sand fly exposure and infection. Plos Neglected Tropical Diseases [Internet]. 2021 Jun;15(6). Available from: ://WOS:000664522600001

23. Magill Alan J., Grogl Max, Gasser Robert A., Sun Wellington, Oster Charles N. Visceral Infection Caused by Leishmania tropica in Veterans of Operation Desert Storm. New England Journal of Medicine. 1993;328(19):1383–7.

24. Matheson A, Williams R, Bailey MS. Cutaneous Leishmaniasis in Royal Marines from Oruzgan, Afghanistan. BMJ Military Health. 2012 Sep 1;158(3):221–4.

25. Myles O, Wortmann GW, Cummings JF, Barthel RV, Patel S, Crum-Cianflone NF, et al. Visceral Leishmaniasis: Clinical Observations in 4 US Army Soldiers Deployed to Afghanistan or Iraq, 2002-2004. Archives of Internal Medicine. 2007 Sep 24;167(17):1899–901.

26. Mody RM, Lakhal-Naouar I, Sherwood JE, Koles NL, Shaw D, Bigley DP, et al. Asymptomatic Visceral Leishmania infantum Infection in US Soldiers Deployed to Iraq. Clinical Infectious Diseases. 2019 May 30;68(12):2036–44.

27. Obwaller AG, Köhsler M, Poeppl W, Herkner H, Mooseder G, Aspöck H, et al. Leishmania infections in Austrian soldiers returning from military missions abroad: a cross-sectional study. Clinical Microbiology and Infection. 2018 Oct 1;24(10):1100.e1-1100.e6.

28. Oré M, Sáenz E, Cabrera R, Sanchez JF, Santos MBDL, Lucas CM, et al. Outbreak of Cutaneous Leishmaniasis in Peruvian Military Personnel Undertaking Training Activities in the Amazon Basin, 2010. The American Journal of Tropical Medicine and Hygiene. 2015 Aug 5;93(2):340–6.

29. Patino LH, Mendez C, Rodriguez O, Romero Y, Velandia D, Alvarado M, et al. Spatial distribution, Leishmania species and clinical traits of Cutaneous Leishmaniasis cases in the Colombian army. PLOS Neglected Tropical Diseases. 2017 Aug 29;11(8):e0005876.

30. Royer M, Crowe M. American Cutaneous Leishmaniasis: A Cluster of 3 Cases During Military Training in Panama. Archives of Pathology & Laboratory Medicine. 2002 Apr 1;126(4):471–3.

31. Sanchez JL, Diniega BM, Small JW, Miller RN, Andujar JM, Weina PJ, et al. Epidemiologic investigation of an outbreak of cutaneous leishmaniasis in a defined geographic focus of transmission. Am J Trop Med Hyg. 1992 Jul 1;47(1):47–54.

32. Van Der Snoek EM, Lammers AM, Kortbeek LM, Roelfsema JH, Bart A, Jaspers CAJJ. Spontaneous cure of American cutaneous leishmaniasis due to Leishmania naiffi in two Dutch infantry soldiers. Clinical and Experimental Dermatology. 2009 Dec 1;34(8):e889–91.

33. van Thiel PP, Leenstra T, de Vries HJ, van der Sluis A, van Gool T, Krull AC, et al. Cutaneous Leishmaniasis (Leishmania major Infection) in Dutch Troops Deployed in Northern Afghanistan: Epidemiology, Clinical Aspects, and Treatment. Am J Trop Med Hyg. 2010 Dec 6;83(6):1295–300.

34. van Thiel PPAM, Zeegelaar JE, van Gool T, Faber WR, Kager PA. Cutaneous leishmaniasis in three Dutch military cohorts following jungle training in Belize. Travel Medicine and Infectious Disease. 2011 May 1;9(3):153–60.

35. Vickery JP, Tribble DR, Putnam SD, McGraw T, Sanders JW, Armstrong AW, et al. Factors Associated with the Use of Protective Measures against Vector-Borne Diseases among Troops Deployed to Iraq and Afghanistan. Military Medicine. 2008 Nov;173(11):1060–7.

36. Wijerathna T, Gunathilaka N, Semege S, Pathirana N, Rodrigo W, Fernando D. Genetic diversity of Leishmania donovani isolates from cutaneous lesions of military personnel in the Mullaitivu and Kilinochchi districts of the Northern Province, Sri Lanka. Asian Pacific Journal of Tropical Medicine. 2022 Sep;15(9):418–24.

37. Woodrow JP, Hartzell JD, Czarnik J, Brett-Major DM, Wortmann G. Cutaneous and Presumed Visceral Leishmaniasis in a Soldier Deployed to Afghanistan. MedGenMed. 2006 Nov 30;8(4):43.

38. Study Quality Assessment Tools | NHLBI, NIH [Internet]. [cited 2023 Jun 23]. Available from: https://www.nhlbi.nih.gov/health-topics/study-quality-assessment-tools

39. The PRISMA 2020 statement: an updated guideline for reporting systematic reviews | The BMJ [Internet]. [cited 2023 Mar 29]. Available from: https://www.bmj.com/content/372/bmj.n71

40. Moher D, Liberati A, Tetzlaff J, Altman DG. Preferred reporting items for systematic reviews and meta-analyses: The PRISMA statement. International Journal of Surgery. 2010 Jan 1;8(5):336–41.

41. Brooke BS, Schwartz TA, Pawlik TM. MOOSE Reporting Guidelines for Meta-analyses of Observational Studies. JAMA Surgery. 2021 Aug 1;156(8):787–8.
