# Supplementary material for: Leishmaniasis in deployed military populations: A systematic review and meta-analysis
Source: PLoS Negl Trop Dis. 2025 Mar 10;19(3):e0012680. doi: 10.1371/journal.pntd.0012680 (PMC11913291; doi:10.1371/journal.pntd.0012680)
Supplement: Attachment — Submitted filename: Response to Reviewers.docx [file pntd.0012680.s003.docx]

**Responses to Reviewers’ comments**

We thank the reviewers for their useful comments, which we have addressed as itemised below. In adding comments to the text, we have restructured the Discussion in places but have not altered the overall synthesis from the submitted manuscript. The estimates have not been altered.

| **Reviewer 1** | |
| --- | --- |
| The review selection criteria for inclusion/exclusion of literature could be improved:  Search strategy and selection criteria: The text states, “Specific subtopics or aspects of leishmaniasis in the military were considered, such as…” How were the subtopics “incidence” (not cumulative) and “prevalence” used in the search and selection criteria? Since cumulative incidence (or attack rate), characterization of sand fly and Leishmania species, sand fly saliva exposure, effectiveness of PPMs, and associated risk are listed as inclusion criteria, but not “incidence” and “prevalence2, I recommend removing "incidence" and "prevalence" from this sentence to avoid confusion. | Thank you for your comments. We have removed the words as suggested to avoid confusion |
| Cumulative incidences of cutaneous leishmaniasis: Pooling incidence data from geographically disparate regions with differing reservoirs, vectors, and parasites raises questions about its validity. Even within the same WHO region, heterogeneity can be extremely high. Conducting a meta-analysis with only three studies also raises concerns—is it valid? Given the high inter-study heterogeneity, what is the rationale behind generating a unified indicator for such disparate data? | This is a valid concern. We considered this question, and acknowledge differences in vectors, reservoirs and parasites in the Discussion, but went on to pool the data for the following reasons:  **Justification for Pooling Data**  Pooling data in this analysis was undertaken to provide an overall estimate of cumulative incidence in a specific subpopulation—deployed military personnel—who share many unique risk factors due to deployment-related activities:   - Shared Exposure Characteristics: Military personnel, irrespective of deployment region, are often exposed to CL through common risk-enhancing factors such as outdoor sleeping conditions, lack of protective measures, and prolonged time spent in vector-rich environments. - Clinical Relevance: A unified indicator helps military health planners identify and prepare for the average burden of disease in deployed troops, which may not be adequately represented by individual studies. - Heterogeneity Management: While heterogeneity was high (I² = 97.84%) as recognised and discussed, subgroup analyses were conducted to explore sources of variability (e.g., region, duration of deployment, and seasonality). This approach provides a nuanced understanding of incidence variations without assuming homogeneity across studies.   Military healthcare systems often operate globally and benefit from generalised metrics to estimate risks, allocate resources, and design prevention programs. Given the lack of extensive studies, generating a unified estimate offers a starting point for guiding policy while highlighting the need for more context-specific research.  See the Discussion (page 22). In addition, we have added a definition of cumulative incidence to the Methods section. |
| Adoption of PPMs and associated risk of developing leishmaniasis: Replace “however the latter two PPMs were not individually significant from controls” with “however the latter two PPMs were not individually significantly different from controls.”  Additionally, please specify which PPMs were used in the studies. Since the PPMs can vary significantly, the effectiveness and associated risk of developing leishmaniasis may depend heavily on the type of PPMs employed. | The sentence has been edited as suggested.  The PPMs used in the studies are already displayed on the forest plot (Figure 5). |
| Symptomatic visceral leishmaniasis (VL): The meta-analysis for cutaneous leishmaniasis (CL) was based on cumulative incidence and odds ratios for developing leishmaniasis associated with PPMs. However, the description of the studies on VL does not address these aspects. Is this because such data were not available in the literature? Why was a meta-analysis of cumulative incidence possible for asymptomatic CL but not for VL, given that both analyses involved three studies? Please clarify. | The key difference between the meta-analysis conducted for CL and the description of VL lies in the availability and nature of the data:   - For CL: The included studies provided numerical data on cumulative incidence and odds ratios (ORs) associated with PPMs. These data enabled meta-analysis to synthesise quantitative outcomes despite heterogeneity. - For VL: The studies described symptomatic cases in narrative form without reporting cumulative incidence rates or ORs. The data on VL were primarily qualitative, focusing on clinical presentations and specific case outcomes rather than broader population-based metrics.   Reasons for providing a narrative analysis only are mentioned in the Methods. A short paragraph has been added in the Symptomatic VL section of the results also to clarify this (page 14). |
| Symptomatic CL: The first two sentences in this section are unnecessary. You should begin by emphasizing the key findings of your review and meta-analysis. | The two sentences have been removed as recommended, and a new opening statement has been added to highlight the key findings. |
| Again, what is the value of generating a unified indicator for certain metrics when heterogeneity is so high? Please address this in the discussion section. Beyond stating the study's limitations, assess how reliable the incidence values are, given the elevated heterogeneity. While this approach may facilitate comparisons between studies, it seems less effective for synthesizing the available scientific information when contexts differ so greatly. You might consider drawing conclusions about this issue. | This has been addressed in the Discussion section, and a conclusion has been drawn (See page 20-21). See also comments above, |
| The higher cumulative incidence (20% [95% CI: 16, 25]) associated with deployments lasting 1–4 months compared to those lasting less than 1 month or more than 4 months (<7%) was not discussed. Please address this finding. | Comments on this observation have been added to the Discussion - page 22. |
| Introduction  Some sentences lack citations, e.g., the last sentences of paragraphs 1 and 2 in the Introduction.  You use "PPM" to refer to "personal protective measures" in the summary but "personal preventative measures" in the Introduction. Please unify the terminology throughout the manuscript. | Done |
| Discussion  Spell out the full names of species when starting a sentence, e.g., “Leishmania donovani, found predominantly in the Indian subcontinent and East Africa…” | Done |
| **Reviewer 2** | |
| The results are very clearly exposed. A few comments:  - page 9: one study reported both CL and VL: please indicate here in which country, as I do not see this study in the Table  -page 11: the duration of deployment lasting 1-4 months appeared to result in a higher cumulative incidence compared to < 1 month (this is understandable) and > 4 months (how do the authors explain this? a duration > 4 months should be associated with a higher risk.  - page 11: screening for Leishmania DNA by PCR: please specify which kind of samples were used, as this can have an important influence on sensitivity (swabs, skin biopsy, scrapings...)  - page 12: the abbreviation LLIN has not been explained | Thank you for your comments.  The country has now been added.  An comment has been added under the Discussion section.  The sample type has been added to the sentence.  The abbreviation is now written in full. |
| **General comments** | |
| Diseases can hardly be fathomed without considering works in French, Spanish and Portuguese for the New World, and Middle East languages for the Old World.  Besides, using papers only in English narrows the scope by focusing on US and UK military populations, when other armies with limited resources could be associated with different results. | Most of the papers identified in other languages had English translations available, which allowed us to include them in our analysis. We had data from US, UK, French, Dutch, German, Brazilian, Sri Lankan, Colombian, Puerto Rican, Peruvian, Austrian, Pakistani, Fijian, Italian and Uruguayan soldiers (Table 1). Consequently, only 9 with no English papers (Russian and Portuguese) were excluded; of which only 2 seem to have potentially relevant data. |
| I think Table 1 should indicate, for each study, the nationality of infected soldiers (when available). The conclusions in terms of immunity and prevention are very different if the infection occurs in Saudi soldiers living in endemic areas (for example) or US soldiers deployed for a short term in Saudi Arabia. | We have now included the nationality of infected soldiers in Table 1. |
| If the authors found no study involving local military populations infected in their own country, this point should be discussed. For example, it is known that outbreaks occur in French-speaking African armies in Western Africa, but these outbreaks are seldom published (and usually in French). | We found five studies involving local military populations infected during deployments within their own country (see Table 1), but only one of these studies provided suitable data for inclusion in our analysis. Findings from the other four studies are captured in the narrative (Table 1) and discussion. |
| Another point that should be discussed (if possible) is the type of activity performed by the military at the time of each outbreak. This should be detailed in Table 1 and discussed. For example, the outbreak in French Guiana in 2020 was linked to intense training in the rainforest and not actual fighting, which can hardly be compared with real frontline situations of US troops in the Middle East. | Thank you for your comment. The type of activity performed by the military at the time of each outbreak has been included in Table 1 and discussed. |
| Page 23, the authors mention the importance of vectors endophagy/exophagy. There are differences in vectors behaviours between each endemic area (due to phlebotomines local ecology). Can these differences explain the discrepancies in the efficiency of PPMs? | Thank you for your comment. Differences in vector behaviour, particularly endophagy versus exophagy, indeed play a critical role in determining the effectiveness of PPMs. Variability in sand fly behaviour across endemic areas, influenced by the local ecology, may explain the observed discrepancies in PPM efficacy. Sand flies are generally considered opportunistic blood feeders; both in their biting behaviours and host choice. However, there is an absence of experimental and/or robust observational studies on sand fly biting behaviour, which limits our understanding. |
| Finally, the authors rightly point the difficult definition of rain or dry season across a large range of heterogenous countries and studies. However, it seems to me that Table 1 suggests that outbreaks mostly occur during wet seasons in the New World and dry season in the Old World, is that in line with usual data in civilian populations? | Yes, indeed the data generally aligns with patterns observed in civilian populations, although these trends are not universally consistent (as addressed in the Discussion- see page 21). |
